# Chemical Conjugation Strategies for the Development of Protein-Based Subunit Nanovaccines

**DOI:** 10.3390/vaccines9060563

**Published:** 2021-05-28

**Authors:** Lantian Lu, Viet Tram Duong, Ahmed O. Shalash, Mariusz Skwarczynski, Istvan Toth

**Affiliations:** School of Chemistry and Molecular Biosciences, The University of Queensland, St Lucia, QLD 4072, Australia; l.lu@uq.edu.au (L.L.); tram.duong@uq.edut.au (V.T.D.); a.shalash@uq.net.au (A.O.S.)

**Keywords:** conjugation strategies, protein subunit vaccines, nanovaccines, vaccine delivery, nanoparticulate delivery systems

## Abstract

The production of subunit nanovaccines relies heavily on the development of a vaccine delivery system that is safe and efficient at delivering antigens to the target site. Nanoparticles have been extensively investigated for vaccine delivery over the years, as they often possess self-adjuvanting properties. The conjugation of antigens to nanoparticles by covalent bonds ensures co-delivery of these components to the same subset of immune cells in order to trigger the desired immune responses. Herein, we review covalent conjugation strategies for grafting protein or peptide antigens onto other molecules or nanoparticles to obtain subunit nanovaccines. We also discuss the advantages of chemical conjugation in developing these vaccines.

## 1. Introduction

Since Edward Jenner’s development of a smallpox vaccine using cowpox in 1798 [1], vaccines have played an important role in the prevention of infectious diseases. Vaccines provide immunity against various pathogens and have also been utilized to combat tumors in cancer immunotherapy [2]. Typically, they are used to stimulate long-term cellular and humoral immunity and induce a proliferation of memory cells to combat future infections or disease recurrence [3]. Vaccines can be grouped into two categories: whole-pathogen vaccines and subunit vaccines.

Whole-pathogen vaccines utilize whole microorganisms, such as viruses and bacteria, which may be live (attenuated or not attenuated) or killed. Vaccines that contain living pathogenic microorganisms are limited by their requirement for refrigerated transport to preserve potency, and the chance that they could revert to their virulent forms under particular conditions [4]. Attenuated vaccines usually do not require refrigeration and are transported in dried form; however, these vaccines induce weaker immune responses, and booster doses may be needed to maintain the potency of immune response [5]. Moreover, traditional whole-pathogen vaccines may induce extensive inflammation, allergies, and autoimmune responses. These drawbacks of traditional vaccines have limited their applications [6]; albeit, traditional vaccines are widely used in veterinary medicines [7]. The safety criteria in veterinary vaccines are much less strict compared to those approved for human use.

Subunit vaccines contain purified or recombinant subunit components derived from a particular pathogen, such as proteins or peptides that have antigenic properties [8,9]. In comparison to their whole-pathogen counterparts, subunit vaccines have minimal adverse effects, do not require complex storage or transport conditions, and have large-scale manufacturing potential [10,11,12]. However, subunit vaccines are often unable to trigger strong immune responses and require the application of nanotechnology or molecular adjuvants to boost the immunity [13,14,15,16].

Adjuvants are designed to trigger immune responses and indirectly activate adaptive immunity. Adjuvants fall into two classes: delivery systems and immunopotentiators [17]. Immunopotentiators can directly activate the innate immune system and are recognized by receptors of antigen-presenting cells (APCs). In contrast, delivery systems protect antigens from degradation while delivering them to specific tissues or cells [18,19]. Adjuvants are often considered to be immunopotentiators. While delivery systems are not considered to be adjuvants per se, they might be imparted with self-adjuvanting properties due to their enhancement in APC uptake, processing, and presentation of antigens.

Shi et al. summarized the mechanisms of how different adjuvants activate the human immune system to induce immune responses [20]. Nanoparticle (NP)-based formulations are one of the most popular drug delivery systems, having been widely investigated over the past two decades [21,22]. Simultaneously, nanotechnology has been developing rapidly for vaccine delivery applications [16,23,24]. These “nanovaccines” utilize NPs in the range of 1–1000 nm, as particles this size can traffic in the lymphatic system and are preferentially taken up by APCs [25]. Various NP-based delivery systems are now under development. These include lipid-based delivery systems, such as liposomes and solid lipid NPs; polymer-based delivery systems, such as chitosan, hyaluronic acid, alginate, poly(lactic-co-glycolic acid), and polypeptide NPs; and inorganic delivery systems, such as gold NPs and silica NPs [19,26,27,28,29,30]. Nanoparticle-based vaccine delivery systems often possess self-adjuvanting properties and, therefore, no additional adjuvants are required to stimulate strong humoral and/or cellular immune responses, such as high induction of antibody titers, long-lived plasma cells, or cytotoxic CD8+ T cells [27,31].

**Figure 1:**
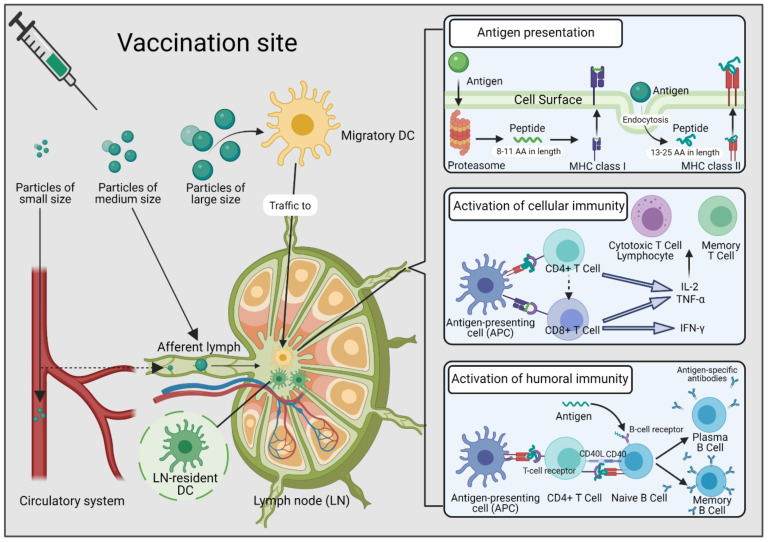
Vaccine particles of different sizes delivered by immune cells to induce Th1 and/or Th2 immunity. Small vaccine particles (normally < 5 nm) hardly drain to the lymph nodes (LNs) and are disseminated into the circulatory system and eliminated; vaccine particles of medium size (5–200 nm) can drain to the LNs directly, while larger particles (normally > 200 nm) require the assistance of migratory dendritic cells (DCs) to delivery antigens to lymph nodes, where they are recognized and internalized for further presentation. Once internalized, antigens are proteolyzed in endosomes or by proteasomes into small peptide fragments. These peptide fragments then bind with major histocompatibility (MHC) class I or II molecules to activate cellular immunity directly or assist in generating humoral immunity through the interaction between CD4+ T cells and B cells. AA, amino acid; APC, antigen-presenting cell; CD40, cluster of differentiation 40; CD40L, cluster of differentiation 40 ligand; DC, dendritic cell; IL-2, interleukin-2; TNF-α, tumor necrosis factor alpha; IFN-γ, interferon gamma. Drawing created with BioRender.com (accessed 26 May 2021).

Immune responses can be modulated by controlling the physicochemical characteristics of antigen-bearing NPs, such as size, shape, and surface charge. NP shape plays a role in the particles’ ability to reach the cytoplasm without being disrupted by endosomes [32] and can also mediate the initiation of phagocytosis by APCs, such as macrophages [33]. NP size determines the way they travel to the lymph nodes, where the immune responses are initiated (Figure 1).

NPs with a size of 5–200 nm can drain to the lymph nodes directly, whereas assistance from migratory dendritic cells (DCs) is required for larger particles [34,35,36]. Nanovaccine-containing particles with a size that allowed for direct trafficking to the lymph nodes showed higher accumulation in lymph node-resident DCs [37]. Size was also found to influence the retention and presentation of nanovaccine in the lymph nodes. When 15, 50, and 100 nm nanoparticles were examined for their distribution in lymph nodes, all of them were taken up by follicular DCs; however, the 15 nm particles were cleared more quickly from the nodes (in days) and then from follicular DCs (through endolysosomal escape and formation of extracellular vesicle). By contrast, larger particles were retained in the lymph nodes for several weeks and induced stronger humoral immune responses [38]. However, the optimal size of nanovaccines for immunization may differ depending on the route of administration. For example, NPs less than 300 nm are the most effective at crossing mucus for intranasal delivery [39].

**Figure 2:**
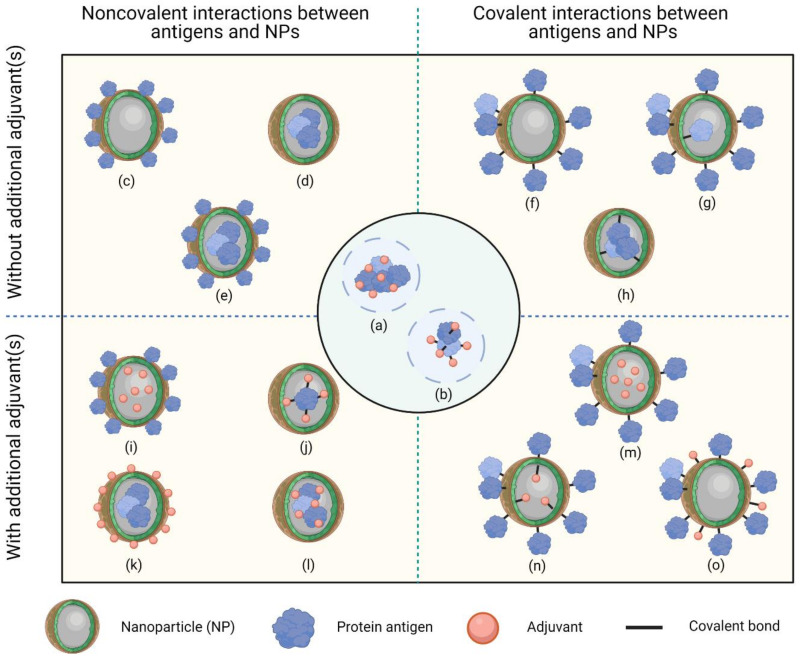
The most common designs of nanoparticle (NP)-based vaccines. Antigens can be associated with NPs via noncovalent interactions (left panels) or covalent interactions (right panels). Additional adjuvants can be incorporated into formulations to elicit stronger immune responses (bottom panels), or NPs can be administered on their own (top panels). (**a**) Antigen physically mixed with adjuvant through electrostatic or hydrophobic interaction. (**b**) Antigen conjugated to adjuvant via covalent bond. (**c**) Antigen coated onto the surface of NPs through electrostatic or hydrophobic interaction (in some cases, there may be multiple coating layers). (**d**) Antigen encapsulated in NPs. (**e**) Antigen coated onto the surface of NPs, as well as encapsulated within NPs. (**f**) Antigen conjugated to the outer surface of NPs. (**g**) Antigen conjugated to both the inner and outer surfaces of NPs. (**h**) Antigen conjugated to the inner surface of NPs. (**i**) Antigen coated onto the surface of NPs, with adjuvant encapsulated in the NPs. (**j**) Antigen–adjuvant conjugate encapsulated in NPs. (**k**) Adjuvant coated onto the surface of NPs, with antigen encapsulated in the NPs. (**l**) Mixture of antigen and adjuvant encapsulated in NPs. (**m**) Antigen conjugated to the surface of NPs, with adjuvant encapsulated in the NPs. (**n**) Antigen conjugated to the outer surface of NPs, with adjuvant conjugated to the inner surface. (**o**) Both antigen and adjuvant conjugated to the outer surface of NPs. NP, nanoparticle. Drawing created with Biorender.com (accessed 26 May 2021).

NP surface charge influences solubility, uptake by APCs, and DC maturation. Positively charged nanovaccines have the propensity to be taken up more efficiently by APCs compared to their neutrally or negatively charged counterparts due to interactions with negatively charged cell surfaces. For example, cationic liposomes with higher charge intensities induced antigen uptake by DCs, DC maturation, and antigen-specific IgG production to a greater extent [40]. However, negatively charged NPs can sometimes also be efficiently taken up by APCs [41,42,43,44]. Negatively charged nanovaccines with a size smaller than 100 nm may have potentially more specific immunomodulatory capacity, as they do not interact with positively charged non-immune cells [45]. The capacity for antigen-associated NPs to elicit immune responses can be strengthened by incorporating targeting moieties on the NP surface to improve their recognition by APCs [46,47,48,49,50]. Various targeting moieties, such as mannose and antibodies, have been used for this purpose for different subsets of APCs [48,49,51,52]. Figure 2 shows the most common designs of NP-based vaccines.

Association between antigens and NPs can be achieved through two different mechanisms, namely, noncovalent interaction, such as adsorption or encapsulation, and covalent interaction, which is also known as a conjugation [53]. Adsorption of antigen to the surface of an NP facilitates interactions between the antigen and surface receptors, such as Toll-like receptors on APCs [26]. Adsorption can be achieved by hydrophobic or electrostatic interactions between antigens and NPs [54]. This process is dependent on factors such as size, shape, surface charge, functional groups, and hydrophobicity of the NPs, as well as the suspending medium [55]. Encapsulation of antigens within a NP can protect the antigens from premature protease degradation and elicit sustainable release [26]. Encapsulation is usually achieved by emulsification [56] or liposome formation [23,57]. Conjugation of antigens to NPs is achieved through chemical reactions that form a covalent bond. Strong association between antigens and NP delivery systems allow these components to be delivered to the same immune cells without dissociation, which is beneficial for triggering immune responses [19,23]. Herein, we review conjugation strategies for the development of protein-based subunit nanovaccines and the advantages and disadvantages of each strategy. This includes approaches used to form covalent bonds between antigens and NPs to obtain protein nanoconjugates. We conclude with a future outlook for protein nanoconjugates. Our review suggests that these strategies will produce promising vaccine candidates capable of eliciting robust immune responses against different diseases.

## 2. Conjugation Strategies to Form Protein Nanoconjugates

Recombinant fusion, chemical conjugation, enzyme-mediated conjugation, photo-conjugation, and various mixtures of these methods have all been investigated for covalently attaching proteins and other molecules [58]. In this section, the most common chemical methods are reviewed.

### 2.1. Conjugation Sites in Proteins

**Figure 3:**
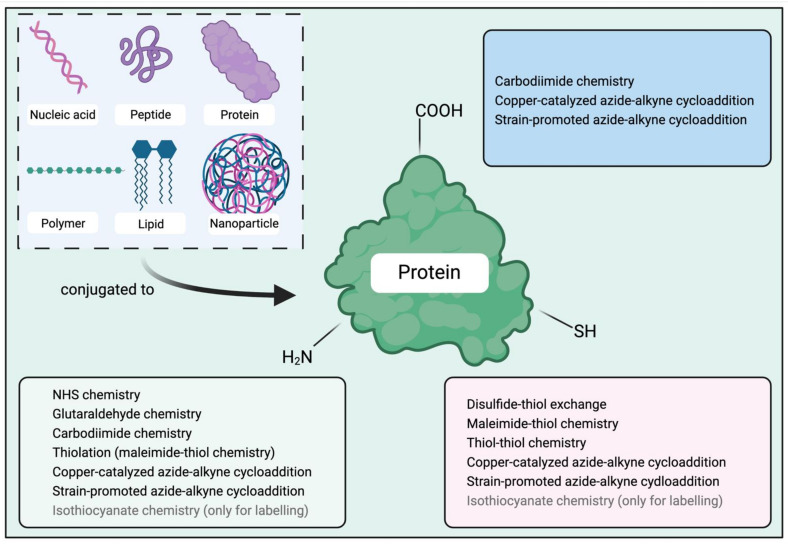
Conjugation strategies targeting different functional groups of proteins. Compounds, such as nucleic acids, peptides, proteins, polymers, lipids, and nanoparticles, can be conjugated to proteins via methods that target the amine, thiol, or carboxyl groups of proteins. NHS, N-hydroxysuccinimide. Drawing created with BioRender.com (accessed 26 May 2021).

For proteins to be grafted with other molecules, side chain functionalities or C- and N-terminal moieties of the proteins are targeted for modifications (Figure 3). Amine groups are ubiquitous in proteins: they exist either at the N-terminus of a protein sequence or at the side chain of lysine residues. As these amine groups are positively charged at their physical pH, they tend to occur on the protein surface, making them accessible for conjugation. However, modification of the amine groups can cause protein structure disruption if too many lysine residues are functionalized [59]. In addition, modification of lysine moieties on the surface of antigens can disrupt their conformational properties. B-cell epitopes can especially be substantially affected, as they are sensitive to amino acid modification, steric hinderance (bulkiness of functionalities attached to lysine), and conformational changes. In comparison with amines, thiols are much less prevalent in proteins, making them a more site-specific target for conjugation. However, thiols are not always accessible on the protein surface, as they usually form disulfide bonds or are not sufficiently exposed for conjugation due to steric hindrance from protein structures. Thiol groups can be artificially generated by application of reducing reagents that break disulfide bonds, or with thiolation reagents that introduce new thiol groups into the protein [60,61,62,63]. Carboxyl groups are the third target for conjugation. They can be found at the side chain of aspartic acids and glutamic acids, or at the C-terminus of a protein. Although a carboxylic acid can react with an amine to form an amide through dehydration/condensation, the reaction is relatively difficult to produce naturally as the alkaline amine group can deprotonate the carboxyl group to form a highly unreactive carboxylate. Thus, coupling of an amine and carboxylic acid involves the use of a carboxylic group activation reagent [64]. Unfortunately, this type of activation can trigger intramolecular reactions of the activated carboxylic group with amine from the same protein or crosslinking of distinct protein molecules.

### 2.2. Conjugation Methods

Chemical conjugation methods are usually designed to modify the amine, carboxyl, or thiol groups of a protein (Figure 3). A variety of molecules have been conjugated to proteins, including nucleic acids, peptides, proteins, polymers, lipids, and NPs. Herein, we present nine popular conjugation methods and discuss their advantages and limitations (Table 1).

**Figure 4:**
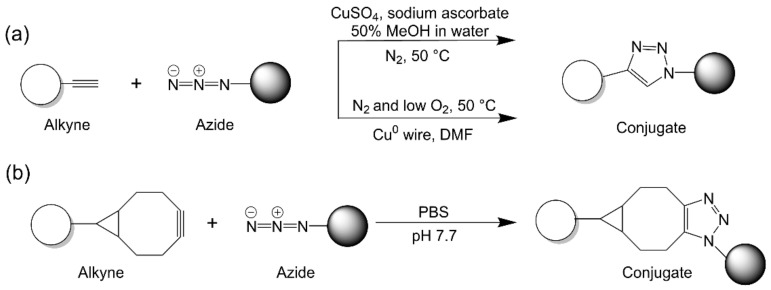
“Click” chemistry. (**a**) Copper-catalyzed azide–alkyne cycloaddition. (**b**) Strain-promoted azide–alkyne cycloaddition.

Copper-catalyzed azide-alkyne 1,3-dipolar cycloaddition (CuAAC) belongs to the family of “click” reactions [65]. CuAAC utilizes copper (Cu^2+^ or Cu^0^, such as copper wire) to catalyze a reaction between an alkyne and an azide group, with sodium ascorbate, hydrazine, or hydroxylamine as the reducing agent (Figure 4a) [65]. However, the use of copper wire as a sole reagent has also been reported [66,67,68,69]. CuAAC is a well-established method that has been extensively used for peptide–peptide or peptide–polymer conjugation [70,71]. The technique can be performed in living cells to modify cellular proteins, but yield from in vivo conjugation is low [72]. The conjugation efficiency of CuAAC reactions can be improved when the copper catalyzer is associated with polymeric NPs [73]. Alkyne and azide groups are not native to biomolecules; therefore, the application of this reaction for conjugation must always involve the introduction of azide and alkyne functional entities for intact biomolecules [65].

As azide and alkyne groups are not reactive to the native functional groups of biomolecules, click chemistry results in high site specificity. However, this method has several limitations [65,74]. Firstly, the protein needs to be functionalized with azide/alkyne moieties with the help of often poorly water-soluble azide and alkyne derivatives. This necessitates assistance from an organic solvent, such as DMSO, DMF, or MeCN, which denatures proteins. The involvement of Cu^2+^ and sodium ascorbate leads to the formation of reactive oxygen species, which are known to degrade amino acids, such as histidine, arginine, cysteine, and methionine [65]. It has been shown that CuAAC can result in the irreversible degradation of viral capsids [75]. As copper ions can cause cellular damage or even death above micromolar concentration [76], careful purification of the final product is needed. However, such purification is not trivial, as copper ions can be chelated by the amino acid residues of proteins [77]. One potential solution to circumvent this conjugation challenge is using the copper-free version of the “click” reaction, strain-promoted azide-alkyne cycloaddition [65].

#### 2.2.1. Strain-Promoted Azide–Alkyne Cycloaddition

Strain-promoted azide–alkyne cycloaddition (SPAAC), also known as copper-free click reaction, is another type of “click” reaction [65,78]. SPAAC was developed to alleviate the need for copper, reducing reagents, and accelerating reagents [79]. In this reaction, a linear alkyne group is replaced with a cyclic analogue to react with azide in a more efficient manner, as a result of the high degree of ring strain of cycloalkyne (Figure 4b) [80]. This method has been used often for protein–NP conjugation and has high efficiency [81,82]. SPAAC proceeds to a greater extent than N-hydroxysuccinimide (NHS) chemistry [82] and can be further accelerated by modulating cycloalkyne structure, azide nature, and the solvent system used [78]. For example, the use of dibenzoazacyclooctyne (DIBAC) or bicyclononyne (BCN) as the cycloalkyne-providing moiety in the acetonitrile-containing aqueous solvent was found to increase reaction speed. It was reported that SPAAC can be completed in just five minutes [83]. Like CuAAC, SPAAC also has high site specificity for protein conjugation, as neither cycloalkyne nor azide exist in native biomolecules. SPAAC also proceeds significantly faster than CuAAC in aqueous solvent systems [78]. The main drawback of using SPAAC for conjugation is the cumbersome synthesis of the cyclooctyne precursors [84].

#### 2.2.2. Carbodiimide Chemistry

**Figure 5:**
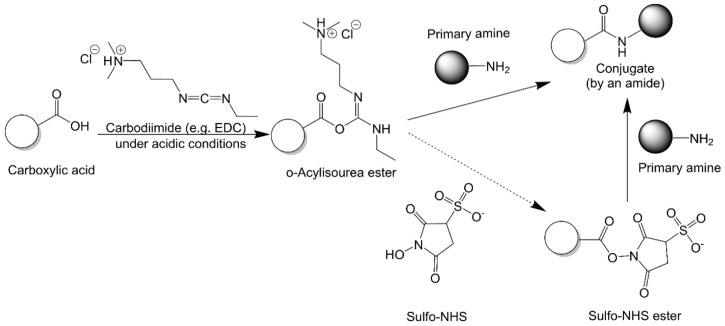
Carbodiimide chemistry. This conjugation reaction can be performed with or without the help of NHS (for example, with the use of the water-soluble derivative of NHS, sulfo-NHS).

Carbodiimides are highly reactive reagents that can activate carboxyl groups to react with amine groups under mild conditions (Figure 5). To date, several carbodiimide molecules, such as 1-ethyl-3-(3-dimethyl-aminopropyl) carbodiimide (EDC) and dicyclohexylcarbodiimide (DCC), have become commercially available. Mechanistically, carbodiimide reacts with carboxylic acid to form an acylisourea intermediate that is subsequently displaced by primary amines to form an amide bond that links the two entities [85]. With a two-step procedure, where NHS is used to convert acylisourea into an NHS ester to further react with amine, conjugation efficiency can be largely improved [86]. Instead of standard NHS, this reaction typically uses sulfo-NHS, as it provides better water solubility. The good water solubility of both EDC and sulfo-NHS allows the conjugation to be performed under aqueous conditions without the use of organic solvents, which add uncertainty to protein stability, as solubility enhancers. Carboxyl group activation is often performed in slightly acidic conditions (pH 6.0 to 6.5) with morpholinoethanesulfonic acid sodium (MES; 0.1 M) buffer and NaCl (0.3 M) as the reaction media [87,88,89]. PBS buffer is also reported to be compatible for carboxyl activation [90]. The main shortcoming of this method is its low efficiency due to competing hydrolysis [81].

#### 2.2.3. N-Hydroxysuccinimide Chemistry

**Figure 6:**
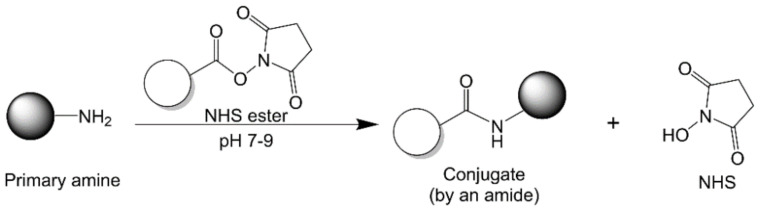
N-hydroxysuccinimide chemistry.

NHS esters can easily react with amines at pH 7–9 (Figure 6), even without carbodiimide pre-activation. However, NHS esters can also react with serine, tyrosine, and threonine hydroxyl residues in proteins, forming undesired conjugation sites [91,92]. Most NHS esters are water-insoluble, and thus they are often introduced to the conjugation media as aliquots in organic solvent, such as DMSO or DMF. However, the involvement of an organic solvent injects uncertainty into protein stability and conformation. Therefore, the amount of organic solvent in the final conjugation media should not exceed a pre-determined percentage (e.g., 10%) in order to preserve the structure and functionality of the proteins [93]. The water solubility of NHS esters can be increased by the inclusion of a charged sulfonate group, as described above [94]. Sodium phosphate buffer (0.1 M) and NaCl (0.15 M) at pH 7.2–7.5 have been recommended as a solvent for this conjugation method [93].

#### 2.2.4. Glutaraldehyde Chemistry

**Figure 7:**
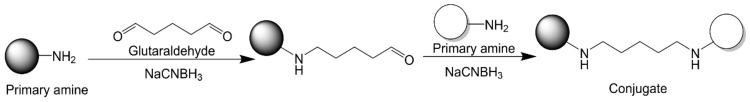
Glutaraldehyde chemistry.

Glutaraldehyde is a homobifunctional crosslinker that is used to join two amine-containing entities together (Figure 7) [95]. Glutaraldehyde first reacts with one amine group by forming a Schiff base, which is highly unstable and easily undergoes reduction to secondary amine. The reaction is then repeated for the other amine moiety. This technique has been favored for its simplicity, speed, and effectiveness for protein conjugation that targets lysine amines or N-terminal amines. However, site specificity can be problematic as cysteine, tyrosine, and histidine are also reactive [96]. Dimers and crosslinking products may also be formed. Another shortcoming of this method is its poor reproducibility. Despite these drawbacks, high conjugation efficiency was reported for NP–protein conjugation using this method under neutral conditions (pH 7.0) [97].

#### 2.2.5. Maleimide–Thiol Chemistry

**Figure 8:**
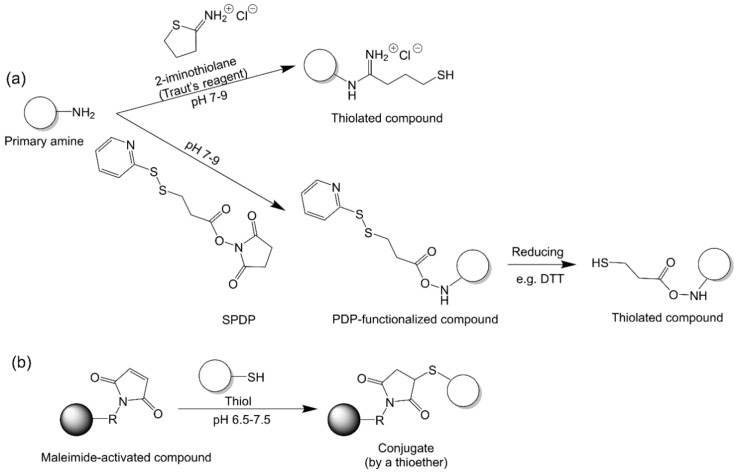
Maleimide–thiol chemistry. (**a**) Thiolation of amine groups. (**b**) Maleimide–thiol reaction.

Maleimides have become one of the most popular choices for achieving site-selective cysteine modifications in proteins [98]. Maleimide–thiol chemistry is favored for conjugation because of its high conjugation efficiency and stability of the conjugates formed. Maleimide specifically reacts with thiols to form a thioether bond at pH 6.5 to 7.5 (Figure 8). However, in more alkaline conditions (pH > 8.5), maleimides also react with primary amines. Thiolation is required if proteins do not carry enough exposed thiol groups on their surface. The most widely used thiolation reagent for this purpose is 2-iminothiolane (Figure 8) [62,63]. N-succinimidyl 3-(2-pyridyldithio)propionate) (SPDP) has also been used to functionalize proteins [35,60]. A reducing reagent, such as dithiothreitol (DTT), is then subsequently employed to reduce the functionalized group into thiol. It was noted that preincubation of maleimides in buffer prior to conjugation led to maleimide hydrolysis, which affected conjugation efficiency [98].

#### 2.2.6. Thiol–Disulfide Exchange

**Figure 9:**
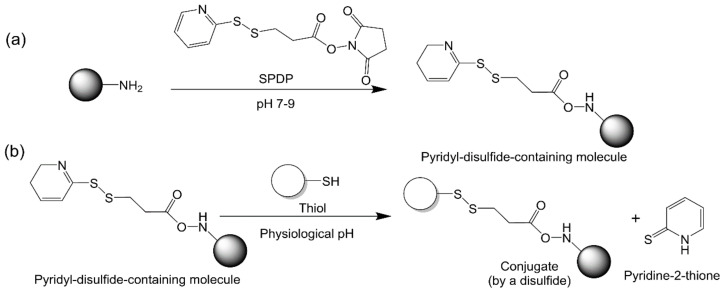
Thiol–disulfide exchange. (**a**) Functionalizing compounds with a pyridydithiol group. (**b**) Thiol–disulfide exchange between a thiol-containing compound and a pyridydithiol-containing compound.

Thiol groups can be exchanged with disulfide-containing molecules to form unsymmetrical disulfides. Pyridyl disulfides have been selected to target cysteine residues for bioconjugation due to their high reactivity with thiols in comparison with other disulfide-containing molecules (Figure 9). Thiol–disulfide exchange can happen under mild conditions: ambient temperature and physiological pH [99]. In addition, this conjugation method can be used for cleavage-on-demand strategies since the reaction is reversible [100].

#### 2.2.7. Tresyl Chloride Activation

**Figure 10:**
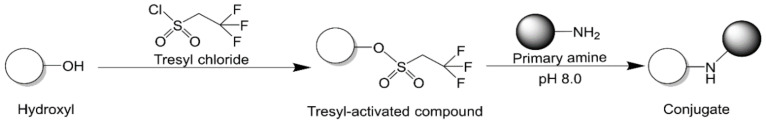
Tresyl chloride activation.

The protein can be also conjugate to the carriers or other moieties through hydroxyl group. In such strategy, hydroxyl group is activated by tresyl chloride to further react with primary amine groups (Figure 10) [101,102,103,104]. Tresyl-functionalized entities are highly reactive with primary amines through alkylation, yielding no spacer between the two compounds. This method has been used to modify adenovirus viral capsid proteins [105,106] and conjugate protein antigens to lipid molecules for liposomal formulations [101,102]. Tresyl chloride activation can be performed in mild conditions [107]. However, as the reaction targets amine groups of biomolecules for conjugation, site-specificity can be a problem in some cases.

#### 2.2.8. Isothiocyanate Chemistry

**Figure 11:**
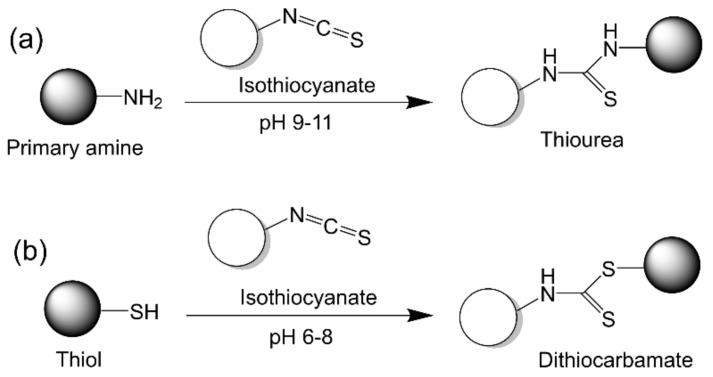
Isothiocyanate chemistry. (**a**) Isothiocyanate reacting with primary amine. (**b**) Isothiocyanate reacting with thiol.

Isothiocyanate (R–C=N=S) can be reactive to both thiol and amine residues of proteins under specific conditions (Figure 11). Under pH 6–8, an isothiocyanate can react with a thiol group to form a dithiocarbamate, while in a more alkaline condition (pH = 9–11), an isothiocyanate preferably reacts with an amine group to form a thiourea [108]. Isothiocyanate chemistry has been widely used for labelling proteins with fluorescent moieties such as fluorescein-5-isothiocyanate (FITC) [109,110,111]. However, this method has not been utilized to produce protein-based subunit nanovaccines yet; thus, it is only presented as a representative method that requires further investigation.

### 2.3. Formation of Protein Nanoconjugates

**Figure 12:**
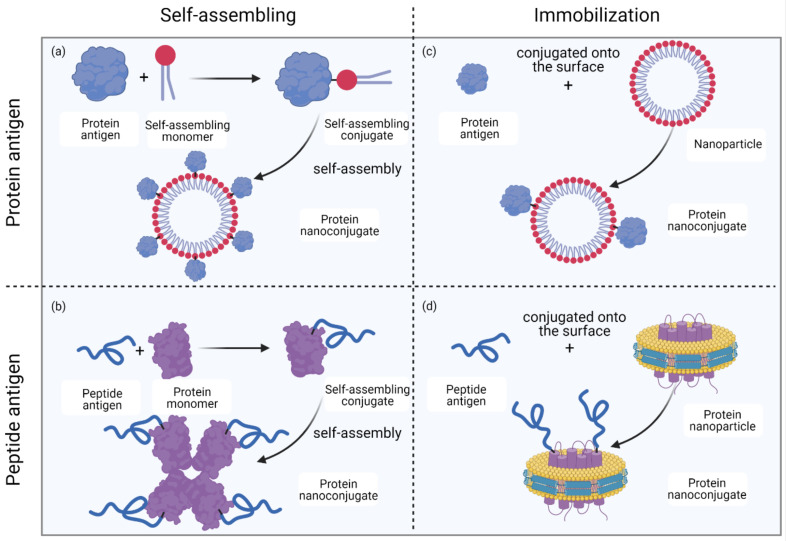
Methods for protein nanoconjugate formation. (**a**) Protein antigens are conjugated to self-assembling monomers; the conjugates then self-assemble into protein nanoconjugates. (**b**) Peptide antigens are conjugated to protein monomers; the conjugates then self-assemble into protein nanoconjugates. (**c**) Protein antigens are conjugated to the surface of nanoparticles (NPs) to form protein nanoconjugates. (**d**) Peptide antigens are conjugated to the surface of NPs (for example, lipoprotein NPs) to form protein nanoconjugates. Drawing created with Biorender.com (accessed 26 May 2021).

Proteins can be conjugated to carrier molecules, such as NPs, or they can be carriers themselves, with smaller antigens conjugated to them (Figure 12). Both conjugations can result in the formation of nanostructures through either self-assembly of formed conjugates, or immobilization when one of the starting materials is pre-formed as NPs. Covalent immobilization can be inhibited by noncovalent associations between NPs and proteins, as adsorption of proteins onto the surface of a NP by hydrophobic or electrostatic interactions occurs in a more spontaneous and rapid manner [86]. Interestingly, noncovalent association of a protein with NPs may prevent its conjugation to NPs, as protein conjugation sites might be shielded by this association.

## 3. Protein Nanoconjugates as Vaccine Candidates

Nanostructures are increasingly popular in the design of protein-based vaccines. NP-associated antigens are more immunogenic than soluble antigens because (a) NPs are preferentially recognized and taken up by APCs since they can mimic the size and some properties of natural pathogens, and (b) presentation of antigens at a high density on the surface of NPs can enhance its recognition by other immune cells such as B cells. Many protein nanoconjugates have shown the ability to elicit strong and effective immune responses.

### 3.1. Protein–Lipid Nanoconjugates

Lipid delivery systems, such as liposomes and lipid nanoparticles (LNPs), have been extensively used in clinics to combat cancer and infectious diseases [112,113,114]. Liposomes are spherical in structure and have a hydrophilic core and one or multiple bilayers of hydrophobic membranes [115]. LNPs can be further categorized into solid lipid nanoparticles (SLN), nanostructured lipid carriers (NLC), lipid drug conjugates (LDC), or polymer-lipid hybrid nanoparticles (PLN). Unlike liposomes, LNPs differ slightly in their individual compositions, as not all LNPs have the continuous bilayer that would qualify them as lipid vesicles or liposomes.

Liposomes have been examined for the delivery of protein antigens for decades. It has been proven that covalent linkage between proteins and liposomes is necessary to induce robust immune responses, as noncovalent protein association (e.g., by chelating) does not produce a product that is stable in vivo [116]. Bale et al. confirmed that noncovalent associations of HIV-1 envelope trimer proteins and liposomes were susceptible to dissociation, while covalent conjugates retained their integrity even after 96 h of incubation at room temperature under in vitro conditions that mimicked the in vivo environment [117]. Although both covalent and noncovalent complexes elicited higher HIV-1 trimer-specific IgG titers in tested animals compared to soluble antigens, covalent HIV-1 trimer–liposome conjugates induced humoral responses to a greater extent compared to their noncovalent counterparts. Notably, mice immunized with covalent conjugates (≈150 nm) had threefold higher germinal center responses and twofold higher antigen-specific T follicular helper cell responses compared to mice immunized with soluble antigens [118].

A variety of other lipidic systems have also been used for protein antigen delivery. For example, Jain et al. conjugated Brij-78 and Brij-700 polymers with protein antigens OVA and HIV p24 via tresyl chemistry [102]. E-wax and water were subsequently added to the conjugates to form a warm oil-in-water microemulsion, which, upon cooling to room temperature, produced SLNs. The nanoconjugates elicited a significantly stronger humoral response in mice compared to monomer conjugates, antigens emulsified in alum, and antigens encapsulated in lipid NPs. Notably, OVA-specific IgG1 titers induced by OVA nanoconjugates (112 nm; +26 mV) were comparable to those of OVA adjuvanted with very strong (but toxic) complete Freund’s adjuvant (CFA).

### 3.2. Protein–Polymer Nanoconjugates

Conjugates of proteins and polymers often form NPs with superior vaccine candidate profiles compared to soluble antigens. Yanase et al. conjugated OVA onto the surface of PEG-functionalized poly(glutamic acid) NPs via maleimide-thiol chemistry [63,119]. The resulting nanoconjugates, which were around 130 nm in size, elicited higher IgG2a and comparable IgG1 titers to OVA emulsified in alum. Interestingly, OVA-specific IgE responses were not produced in the animals tested with OVA conjugated onto the NP surface. In contrast, soluble OVA stimulated an allergic response. Liu et al. conjugated H1N1 antigens onto the surface of N-trimethylaminoethylmethacrylate chitosan (TMC) NPs using maleimide–thiol chemistry [120]. Following intranasal administration to mice, the nanoconjugates (139 nm; +10 mV) induced higher antigen-specific IgG titers in the serum, as well as antigen-specific IgA titers in the nasal tissues, lungs, and saliva compared to soluble antigens, a physical mixture of antigens and NPs; antigen-encapsulated NPs; and alum-precipitated with antigens. In a separate study by the same group, TMMC-OVA nanoconjugates also showed higher transport efficiency to both superficial and deep cervical lymph nodes compared to their physical mixture counterpart following nasal immunization [62]. Notably, the uptake of the nanoconjugates by monocyte/macrophage-like cells, RAW 264.7 cells, was two times higher than that of the physical mixture. Wilson et al. conjugated OVA to poly(mannose-TLR7a) through strain-promoted azide–alkyne cycloaddition [121]. This co-polymer delivery system (710 nm) targeted DCs through both mannose and TLR receptors. Interestingly, nanoconjugates that lacked either mannose or TLR7a elicited lower magnitude of immune responses compared to the co-polymer-conjugated antigens.

Plebanski et al. conjugated malarial protein antigen MSP4/5 to 40–50 nm carboxylated polystyrene NPs that elicited IFN-γ and antibody production with comparable or higher titers than those of MSP4/5 adjuvanted with alum or CFA [122]. Stano et al. conjugated OVA onto the surface of pluronic-stablized poly(propylene sulfide) (PSS) NPs via disulfide bond [123,124]. When co-delivered with CpG, the nanoconjugates (30 nm) induced significantly stronger CD8^+^ T-cell responses compared to antigen-loaded NPs or a mixture of antigen-loaded NPs and nanoconjugates carrying the same number of antigens in total [123]. This delivery system showed excellent characteristics for inducing both protective memory cytotoxic T-cell and T-helper cell responses [125]. When co-delivered with CpG, the uptake of the nanoconjugates by APCs was significantly enhanced; it was sixfold higher than that of soluble OVA with CpG. Ten times more OVA-specific effector CD8^+^ T-cells were detected in the lungs of mice following immunization with nanoconjugates. These enhancements contributed to twofold and ninefold higher IFN-γ and IL-17A titers, respectively, in the animals tested when compared to OVA.

Kim et al. conjugated OVA to nanomaterial hyaluronic acid to enhance the immune response elicited by OVA, as hyaluronic acid receptor-mediated endocytosis can contribute to the enhancement of DC maturation [126]. Although not treated as a nanoconjugate, this conjugate induced 20-fold higher OVA-specific IgG titers in serum than the physical mixture of OVA and polymer when administered intramuscularly. Flanary et al. conjugated OVA to a pH-sensitive polymer, poly(propylacrylic acid), which is a nanomaterial, through disulfide–thiol exchange [127]. Notably, internalization of the conjugates (not necessarily nanoconjugates) by macrophages was more than sixfold higher than that of soluble antigens or the physical mixture of parent compounds. Over five times the amount of nanoconjugate remained in RAW 264.7 cells after 4 h compared to soluble antigens or physically mixed antigens and polymers. A possible mechanism of this delivery system in preventing the exocytosis of antigens in macrophages is that the polymers may disrupt endosomal membranes, which keeps the antigens in the cytosol before exocytosis occurs. Wilson et al. conjugated OVA to an endosome-disruptive polymer via covalent bond through thiol–disulfide exchange [128]. The nanoconjugates (25 nm) electrostatically complexed with CpG enhanced CD8^+^ T-cell responses 7-, 8-, and 18-fold in comparison to immunization with conjugates alone, physical mixture of the three components, and OVA administered with CpG, respectively.

### 3.3. Protein–Protein or Protein–Peptide Nanoconjugates

Proteins in vaccine formulations can act not only as antigens, but also as carriers. Proteins’ biodegradability, biocompatibility, lack of toxicity, and size make them valuable materials for the delivery of biomolecules [129]. When utilized as antigen carriers, many proteins, especially those that are pathogen-derived, can boost the magnitude of immune response elicited by antigens as they comprise many T-helper cell epitopes.

Caged protein NPs consisting of self-assembling protein subunits that form nanocapsules with homogenous size distribution are often used for drug delivery [130]. Chimeric proteins have been widely used to incorporate antigens of interest in cage protein NPs. These systems have typically been produced by a DNA template encoding both of the components [131,132,133,134]; however, conjugation of antigens onto the surface of caged protein NPs through chemical methods has also been investigated. E2 protein can self-assemble into NPs (25 nm) that consist of 60 identical monomers. Maleimide–thiol chemistry has been extensively employed to conjugate epitopes to the external surface of E2 protein NPs [135,136,137]. Interestingly, it was found that not only antigen, but preferably also adjuvant, should be conjugated to the NPs, as both antigen uptake and cross-presentation by DCs were significantly enhanced when CpG molecules were conjugated to the internal surface of E2 protein NPs [135]. It is also worth noting that the dose of CpG needed to activate bone marrow-derived DCs could be reduced to 1/25 when CpG was conjugated to the internal NP surface [137].

Jones et al. conjugated malarial protein antigens with carrier exoprotein A (EPA) via maleimide–thiol chemistry to induce humoral responses in mice [138]. Mice vaccinated with the nanoconjugates (ranging from 14~24 nm) elicited significantly higher antigen-specific antibody titers compared to those vaccinated with monomeric antigens. Similarly, three malarial protein antigens were conjugated to different protein carriers, including EPA, tetanus toxoid, and CRM197, using different conjugation methods to form nanoconjugates with different sizes, ranging from 16 to 73 nm [139]. Notably, the resultant immune response was significantly enhanced when they were conjugated to protein carriers using maleimide–thiol chemistry, NHS chemistry, or adipic acid dihydrazide (ADH)-mediated coupling (a method that targets aldehydes), but not glutaraldehyde chemistry. The authors of the study did not propose an explanation as to why glutaraldehyde chemistry yielded conjugates with lower resultant immune response; however, it may have been a result of the greater extent of intramolecular crosslinking in this method.

Moon et al. conjugated tumor-specific neoantigens onto the surface of a nanodisc produced on the basis of synthetic high-density lipoproteins through thiol–disulfide exchange for personalized cancer immunotherapy [140,141,142,143]. Each nanodisc molecule was able to harbor six peptide antigens via covalent bond, with one CpG molecule encapsulated. Antigen presentation by DCs from this vaccine formulation was ninefold and fourfold higher than that of free peptide antigens with CpG after 24 h and 48 h of incubation, respectively [142]. Together with CpG as an additional adjuvant, the lipoprotein nanoconjugates (10.5 nm) bearing a variety of peptide antigens were able to elicit more than 30-fold greater frequency of antigen-specific CD8α^+^ T cells than peptide antigens mixed with cholesterol-modified CpG.

Good et al. conjugated group A streptococcus (GAS) peptide antigen J8 to diphtheria toxoid (DT) as a carrier protein using maleimide–thiol chemistry to form a peptide–protein conjugate [144,145]. However, the vaccine elicited much higher IgG titers against DT than for J8 in clinical trials, showing the risk associated with the use of protein carrier systems [146]. In addition, DT-based conjugates have been used for screening SARS-CoV-2 spike protein-derived peptide epitopes for the development of a potential peptide-based SARS vaccine by the same group [147].

Valdes-Balbin et al. conjugated fused SARS-CoV-2 RBD with an additional Cys538 to highly immunogenic carrier protein tetanus toxoid through maleimide–thiol chemistry [148]. A higher level of virus neutralization was achieved by the antibody elicited by the conjugates absorbed on alum, compared to RBD/alum. A vaccine based on these conjugates has entered phase II clinical trials in Cuba.

Although some of the protein conjugates mentioned in this chapter were not tested as nanoconjugates, as their size was not examined, it has been demonstrated that conjugating antigens to a protein carrier can be beneficial. With the help of nanotechnology, vaccine design using this strategy is expected to be further improved in the future.

### 3.4. Protein–Inorganic Substance Nanoconjugates

In addition to the organic carriers discussed above, which are composed of biological or biodegradable materials, non-degradable NPs, such as gold, carbon, and silica NPs, have also been investigated for the delivery of protein antigens [149]. Gold nanoparticles (AuNPs) have been recognized as promising vaccine carriers due to their relatively high biocompatibility, preferable size range (typically 10–50 nm for vaccine carriers), homogenous size (very low PDI), stability, and high surface area. Protein antigens have often been complexed with AuNPs via electrostatic interactions or metal chelating [150,151,152,153,154]; however, only a few studies claimed that covalent bonds were formed between AuNPs and protein antigens. Kang et al. conjugated genetically modified OVA (with two cysteine residues at the C-terminus) with AuNPs directly via an Au–S covalent bond [155]. This study demonstrated that the preferable size for AuNPs to induce potent cellular responses including T-cell poly-functionality is between 10 and 22 nm. Gregory et al. conjugated *Yersinia pestis* F1-antigen onto the surface of carboxyl-functionalized AuNPs (15 nm) via carboxyl–amine condensation [156]. AuNP-conjugated F1 antigens induced higher antigen-specific IgG2a titers compared to soluble F1. However, the NPs were unable to induce antibody responses stronger than antigen mixed with alhydrogel adjuvant.

Owing to their mesopores, high surface area, large pore volume, easy modification of physical and chemical functionalities, biocompatibility, and self-adjuvanticity, mesoporous silica nanoparticles (MSNs) are considered to be one of the most promising inorganic nanocarriers [157]. Thalhauser et al. proved that covalent conjugates of MSNs and antigens were able to elicit stronger immune responses compared to their noncovalent counterparts in a study where they conjugated HIV-1 envelope trimers onto the surface of MSNs via maleimide–thiol chemistry [158]. The covalent conjugates (240 nm) enhanced the uptake of antigens by bone marrow-derived dendritic cells (BMDCs) by 4.5-fold, while noncovalent complexes formed via electrostatic interaction only enhanced antigen uptake by 1.4-fold compared to soluble antigens.

Carbon nanotubes (CNTs) belong to another important class of inorganic NPs. Their unique properties, such as inertness, biodegradability, non-immunogenicity, and non-toxicity, have enabled CNTs to be developed as safe vaccine scaffolds [159]. CNTs can be divided into two categories depending on their number of layers: single-walled CNTs (SWCNTs) and multi-walled CNTs (MWCNTs). SWCNTs comprise a single graphene cylinder, while MWCNTs comprise multiple coaxial cylinders [159]. Recently, SWCNTs have been extensively studied as carriers to harbor subunit components for the development of fish vaccines [160,161,162,163,164]. Jia et al. conjugated a recombinant protein antigen to oxidized SWCNTs (1–2 nm in outside diameter and 0.4–3 µm in length) via carbodiimide chemistry [164]. The resulting conjugates elicited significantly higher antigen-specific antibody titers in fish serum compared to antigens alone, increasing immune protection by 53–80%. Additionally, an eightfold lower dose of the conjugate was able to provide a similar extent of protection to the antigen alone.

Less investigation has gone into MWCNTs as vaccine carriers. Oxidized MWCNTs with surface carboxyl groups were conjugated to a tumor cell lysate protein via carbodiimide chemistry by Meng et al. [165]. The conjugates (average diameter: 20–30 nm), administered with inactivated tumor cells, induced a stronger CD8^+^ immune response compared to protein antigens alone. However, no comparison was made for the conjugates alone in inducing cellular immune responses. Further investigation into the adjuvanting properties of MWCNTs is needed.

## 4. Conclusions

Nanoparticulate delivery systems can help to elicit stronger immune responses against antigens by modulating their entry into APCs and/or by making them more recognizable to B cells. Depending on their physiochemical properties, such as size, charge, and morphology, the mechanisms of NP-assisted antigen delivery to APCs or lymph nodes can be different. Antigens can be incorporated into/onto NPs using different strategies. When a covalent bond is formed between a NP and an antigen, it is more difficult to disassociate the antigen from the NP in vivo in comparison to noncovalent complexes. Consequently, covalent bonding facilitates the co-delivery of antigens and NPs to the same immune cells, which enhances the immune response.

Different conjugation methods, targeting different functional groups, have been developed and employed to produce conjugated NPs. Among these methods, strain-promoted alkyne-azide cycloaddition and maleimide–thiol chemistry are the most commonly used synthetic strategies to form nanoconjugates by means of self-assembly. The advantages of these strategies include high conjugation efficiency and site-specificity. However, the application of strain-promoted alkyne–azide cycloaddition often requires expensive and labor-extensive synthetic cycloalkyne compounds, thereby limiting the use for industrial production. Carbodiimide chemistry and copper-catalyzed azide–alkyne cycloaddition have been widely used to form nanoconjugates of inorganic NPs such as AuNPs and organic polymeric NPs, respectively, by the means of surface immobilization. Functionalization of NPs for conjugation is often required for this purpose. It is noted that noncovalent interactions between NPs and antigens such as hydrophobic or electrostatic interactions can interfere with covalent binding. Some conjugation strategies might not be compatible with antigen grafting on the NPs, considering the charge of NPs and antigens, as well as steric hinderance. Whether a conjugation strategy is good or not for a specific purpose needs to be validated in a specific case. A linker between NPs and antigens is formed using most of the conjugation methods given in this review, except for tresyl chloride activation. These linkers are normally too small to induce immune responses against themselves. While introduction of the linker should not affect conjugate antigenicity, some epitopes from the antigen can be disrupted during functionalization or conjugation (e.g., epitopes containing lysine residues in carbodiimide method). It has been noticed that conjugation conditions might influence the stability of nanoparticles, proteins, and/or linkers (e.g., maleimide functional groups), and thus special attention to reaction parameters should be given during conjugation. In sum, there is no universal answer to which conjugation method is most favorable and universal, as the properties of different nanoparticulate delivery systems and protein antigens vary between each other.

Importantly, some antigen-conjugated NPs can induce robust immune responses without the use of additional immunopotentiators, such as TLR agonists. However, many of them still require the use of external adjuvant for optimal efficacy. Still, there are clear advantages of these delivery systems compared to non-conjugated strategies. As the use of NP-based vaccine delivery systems becomes increasingly popular, we can expect further development in protein–NP conjugation techniques, as well as greater application in vaccine designs.

While not necessarily defined as nanovaccines, some protein-conjugated vaccines have already been approved for mass immunization, including PRP conjugate vaccines consisting of polyribosyl ribitol phosphate and various other protein carriers, as well as typhoid conjugate vaccines (Typbar-TCV^®^ and PedaTyph^TM^) consisting of Vi polysaccharide antigens and tetanus toxoid proteins. It can be predicted that with advances in nanotechnology, the promising features of protein-conjugated nanovaccines will be further leveraged to help bridge the gap between the increasing need for vaccines to prevent and cure various diseases and the insufficiencies of current vaccine efficacy and safety.

## Figures and Tables

**Table 1:** Advantages and limitations of protein conjugation methods.

Technique	Advantages	Limitations
Copper-catalyzed azide-alkyne cycloaddition	High site specificity Relatively high conjugation efficiency	Involvement of toxic copper as a catalyst
Strain-promoted azide-alkyne cycloaddition	High site specificity Rapid reaction High conjugation efficiency	Tedious cycloalkyne functionalization
Carbodiimide chemistry	Easy operation Relatively high conjugation efficiency (if NHS is used)	Relatively low site specificity Low conjugation efficiency (if NHS is not used)
N-hydroxysuccinimide (NHS) chemistry	Easy operation Solubility can be increased if a sulfo group is incorporated in the NHS molecule	Relatively low site specificity Water-insoluble (if a sulfo group is not incorporated)
Glutaraldehyde chemistry	Easy operation	Low site specificity Low conjugation efficiency Requirement for toxic NaBH_3_CN as a reducing reagent
Maleimide–thiol chemistry	Easy operation Rapid reaction Relatively high site specificity	Thiolation required (if no thiols are accessible)
Thiol-disulfide exchange	Relatively easy operation Conjugates cleavable in vivo Reaction can be performed at ambient temperature and physiological pH	Thiolation required (if no thiols are accessible) Conjugates cleavable in vivo
Tresyl chloride activation	High conjugation efficiency Reaction can be performed in mild conditions	Relatively low site specificity
Isothiocyanate chemistry	Extensively used for covalent labelling Good water solubility High reaction kinetics at ambiant conditions	Not applied to graft protein antigens with other molecules
